# Antibacterial effect of cell-free supernatant fraction from *Lactobacillus paracasei* CH88 against *Gardnerella vaginalis*

**DOI:** 10.1038/s41598-022-08808-7

**Published:** 2022-03-19

**Authors:** Eun Chae Moon, Myeong Soo Park, Taehwan Lim, Ryun Hee Kim, Geun Eog Ji, Sun Young Kim, Keum Taek Hwang

**Affiliations:** 1 Department of Food and Nutrition, and Research Institute of Human Ecology, Seoul National University, Seoul, 08826 Korea; 2 BK21 FOUR Education and Research Team for Sustainable Food & Nutrition, Seoul National University, Seoul, 08826 Korea; 3 Research Center, BIFIDO Co., Ltd, Hongcheon, 25117 Korea; 4 Department of Biomedical Engineering, Tufts University, 4 Colby St., Medford, MA 02155 USA

**Keywords:** Antimicrobials, Bacteria, Pathogens

## Abstract

Bacterial vaginosis (BV) is the most common vaginal infection in reproductive women, which is characterized by depleted level of lactic acid bacteria and overgrowth of anaerobes such as *Gardnerella vaginalis* spp. Lactic acid bacteria have been known to be beneficial for amelioration of BV, since they produce antimicrobial substances against *G. vaginalis* spp. The objectives of this study were to characterize different fractions of cell-free supernatant of *Lactobacillus paracasei* CH88 (LCFS) and investigate antibacterial activity of the LCFS fractions against *G. vaginalis *in-vitro and in-vivo. Antibacterial activity of the LCFS was stable during thermal treatment up to 120 °C for 30 min and maintained at pH ranging from 3.0 to 13.0 except pH 5.0. Fraction below 3 kDa of the LCFS partially lost its antibacterial activity after treatment with proteolytic enzymes. Precipitated protein fraction below 3 kDa of the LCFS (< 3 kDa LCFSP) inhibited the growth and biofilm formation of *G. vaginalis*. Treatment of *L. paracasei* CH88 or the < 3 kDa LCFSP attenuated *G. vaginalis*-induced BV in mice by inhibiting the growth of *G. vaginalis*, reducing exfoliation of vaginal epithelial cells, and regulating immune response. These results suggest that *L. paracasei* CH88 may have potential in ameliorating *G. vaginalis*-induced BV.

## Introduction

Bacterial vaginosis (BV) is the most prevalent vaginal infection in women of reproductive age^1^. Over 3 million women undergo BV annually in the United States^2^. BV is linked with increased risk of preterm birth, miscarriage, acquisition of human immunodeficiency virus, and sexually transmitted infections^3–6^. BV is commonly characterized by reduced level of lactobacilli, elevated vaginal pH, vaginal discharge, vaginal fluid fish odor, and overgrowth of pathogenic anaerobic bacteria such as *Gardnerella vaginalis* spp., *Prevotella bivia*, and *Fannyhessea vaginae*^7^.

*G. vaginalis* spp. are facultative anaerobes detected in up to 95% of BV cases^8^. *G. vaginalis* spp. have been regarded as one of the bacterial consortiums inducing BV that was best-studied species among the pathogenic anaerobic bacteria^9,10^. These bacteria adhere to vaginal epithelial cells and make an environment favorable for growth of other pathogenic anaerobes forming biofilm, leading to an induction of cytotoxicity on vaginal epithelial cells^11^. However, despite over 60 years of research, a definite etiology of BV has not been fully elucidated^12^.

The administration of antibiotics such as clindamycin and metronidazole has been recommended for treatment of BV^6^, since it showed 75–86% cure rates^13^. However, the use of antibiotics has limitations due to their side effects. About 52% of the patients with BV who were orally treated with metronidazole were associated with gastrointestinal complaints^14^. About 80% of the women with BV who were administered with clindamycin were reported to be exposed to pathogenic anaerobes which are resistant to this antibiotic^15^. Moreover, about 60% of women experienced recurrence of BV after 12 months of metronidazole treatment^16^.

To avoid the side effects of antibiotic treatments, there has been an increasing interest in administration of probiotic bacteria, instead of antibiotics, which can alleviate BV. Reid et al. demonstrated that vaginal microbial microbiota in the patients with BV were reinstated after oral administration of *Lactobacillus rhamnosus* GR-1 and *L. fermentum* RC-14, which did not cause any adverse effect^17^. Moreover, oral administration of *L. rhamnosus* GR-1 and *L. fermentum* RC-14 caused a reduction in the levels of pathogenic bacteria and yeasts in vagina^18^. Intravaginal administration of *L. rhamnosus* GR-1 and *L. fermentum* RC-14 to the patients with BV suppressed recurrence of BV^19^. Intravaginal administration of *L. crispatus* CTV-05 to the patients with BV resulted in this bacterium colonizing the vagina, consequently restoring healthy vaginal microbiota in the patients^20^.

Although some reproductive women maintain a healthy vaginal environment without lactobacilli^21^, lactobacilli are still considered as the most common bacteria in vaginal tract of healthy reproductive-age women and play an important role in maintaining healthy vaginal mucosa^22^. Lactobacilli have been known to have protective effects against BV via production of H_2_O_2_ and lactic acid^23–25^. Moreover, they can produce antimicrobial compounds that inhibit growth of pathogens in the urogenital ecosystem^26^. Bacteriocin produced by *L. acidophilus* 160 had inhibitory activity against *G. vaginalis*^27^.

We found that cell-free supernatant of *L. paracasei* CH88 possessed antibacterial activity against *G. vaginalis*. Moreover, cell-free supernatant of *L. paracasei* CH88 that was adjusted to pH 7.0 (LCFS) also showed the antibacterial activity against *G. vaginalis*.

Thus, we hypothesized that the antibacterial activity of the LCFS (pH 7.0) may be attributed to antibacterial compounds other than lactic acid. The aim of this study was to characterize and fractionate the LCFS and to determine antibacterial effects of the LCFS fraction against *G. vaginalis *in-vitro and ameliorative effect on *G. vaginalis*-induced BV in mice.

## Materials and methods

### Bacterial strains and culture conditions

*G. vaginalis* KCTC 5097 was purchased from Korean Collection for Type Cultures (Jeongeup, Korea). *G. vaginalis* was inoculated at 1% (v/v) in brain heart infusion (BHI; Becton, Dickinson and Company (BD), Franklin Lakes, NJ, USA) broth supplemented with 1% (w/v) yeast extract (BD), 0.1% (w/v) maltose (Sigma-Aldrich Co., St. Louis, MO, USA), 0.1% (w/v) glucose (Sigma-Aldrich Co.), and 10% (v/v) horse serum (Thermo Fisher Scientific, Waltham, MA, USA), named as BHIS broth. *G. vaginalis* was cultured at 37 °C with 5% CO_2_ for 18 h and stored in 50% (v/v) glycerol at − 80 °C until further use.

*L. paracasei* CH88 was cultured in BIFIDO Co., Ltd. (Hongcheon, Korea). Briefly, *L. paracasei* CH88 was inoculated at 10% (v/v) in de Man, Rogosa, Sharpe (MRS; BD) broth in a fermentor (LiFlus GX, Hanil Science Co., Ltd., Daejeon, Korea) and was cultured under anaerobic condition (85% N_2_, 10% H_2_, and 5% CO_2_). The culture was kept at pH 5.5 and 37 °C with 150 rpm agitation for 15 h.

### Preparation of the LCFS

Culture broth of *L. paracasei* CH88 was centrifuged at 10,000 × g for 30 min at 4 °C. After centrifugation, the supernatant was adjusted to pH 7.0 with 5 M NaOH and filtered using a sterilized bottle-top vacuum filter (0.22 μm; Merck, Darmstadt, Germany). The filtrate (LCFS) was stored at − 80 °C for further experiments. Prior to every experiment, we confirmed that no colony was observed when the LCFS was spread and incubated on MRS agar plate at 37 °C for 24 h.

## Characterization of the LCFS

### Thermal treatment and pH adjustment of the LCFS

To determine thermal stability on antibacterial activity of the LCFS against *G. vaginalis*, *G. vaginalis* was inoculated at 1% (v/v) in a 96-well plate with the BHIS broth containing 5% (v/v) LCFS heated for 30 min at 60, 80, 100, or 120 °C. *G. vaginalis* was inoculated in the BHIS broth containing 5% (v/v) MRS broth as control. The plate was incubated at 37 °C for 24 h and its optical density (OD) was measured at 600 nm using a microplate reader (SpectraMax 190, Molecular Devices, San Jose, CA, USA) under anaerobic condition (85% N_2_, 10% H_2_, and 5% CO_2_).

To evaluate the effect of pH on antibacterial activity of the LCFS against *G. vaginalis*, pH of the LCFS was adjusted to 3.0, 5.0, 7.0, 9.0, 11.0, or 13.0 with 5 M NaOH or 5 M HCl. *G. vaginalis* was inoculated at 1% (v/v) in a 96-well plate with the BHIS broth containing 5% (v/v) LCFS with different pH. Antibacterial activity of each sample was examined as described above.

### Size-exclusion filtration of the LCFS

To determine molecular weight of the active fraction, Amicon Ultra-15 Centrifugal Filter (Merck) with molecular weight cut-offs (MWCO) of 3, 10, 30, 50, and 100 kDa was used. Fractions of over 100 kDa, 50–100 kDa, 30–50 kDa, 10–30 kDa, 3–10 kDa, and below 3 kDa were obtained according to the manufacturer’s instruction. *G. vaginalis* was inoculated at 1% (v/v) in a 96-well plate with the BHIS broth added with 5% (v/v) LCFS fractions with different molecular weights. The plate was incubated at 37 °C for 24 h and its OD was measured at 600 nm under anaerobic condition (85% N_2_, 10% H_2_, and 5% CO_2_). The fraction below 3 kDa of the LCFS (< 3 kDa LCFS), which had antibacterial activity, was used for further study.

### Proteolytic enzyme treatment on the < 3 kDa LCFS

Proteolytic enzymes were treated on the < 3 kDa LCFS, according to the method of Lee et al. and Mun et al.^27,28^. Proteinase K (MGmed, Seoul, Korea) in 5 mM ethylenediaminetetraacetic acid containing 0.5% (w/v) sodium dodecyl sulfate solution, protease (EC 3.4.21.62, type VIII, Sigma-Aldrich Co.) in 50 mM hydroxymethylaminomethane-hydrochloric acid (Tris–HCl) buffer (pH 7.5), trypsin (EC 3.4.21.4, type II, Sigma-Aldrich Co.) in sterilized distilled water (pH 7.0), pepsin (EC 3.4.23.1, Sigma-Aldrich Co.) in 50 mM citrate buffer (pH 2.0), and α-chymotrypsin (EC 3.4.21.1, Sigma-Aldrich Co.) in 50 mM Tris–HCl/10 mM CaCl_2_ buffer (pH 7.5) were used. All enzyme reactions were performed in the < 3 kDa LCFS at a final enzyme concentration of 2 mg/mL. Proteinase K was incubated at 55 °C for 8 h and inactivated at 95 °C for 30 min. α-Chymotrypsin was activated at 25 °C for 6 h. The other enzymes were incubated at 37 °C for 6 h. *G. vaginalis* was inoculated in a 96-well plate with the BHIS broth added with 5% (v/v) < 3 kDa LCFS treated with different proteolytic enzymes. *G. vaginalis* was inoculated at 1% (v/v) in the BHIS broth containing 5% (v/v) fraction below 3 kDa of MRS broth as control. The plate was incubated at 37 °C for 24 h and its OD was measured at 600 nm under anaerobic condition (85% N_2_, 10% H_2_, and 5% CO_2_).

### Protein precipitation and plate count

The LCFS was mixed with ammonium sulfate (70%, (w/v); Samchun Pure Chemicals, Pyeongtaek, Korea), followed by stirring the mixture for 12 h at 4 °C. The mixture was centrifuged at 10,000 × g at 4 °C for 1 h. Precipitated proteins were suspended with phosphate buffered saline (PBS) and then dialyzed for 48 h using Pur-A-Lyzer Mega Dialysis Kit (Sigma-Aldrich Co.) with a MWCO of 1 kDa in distilled water at 4 °C. Collected proteins were filtered using a 3 kDa MWCO Amicon Ultra Centrifugal Filter to obtain protein fraction over 3 kDa of the LCFS (> 3 kDa LCFSP) and protein fraction below 3 kDa of the LCFS (< 3 kDa LCFSP). The fractionated protein fractions were lyophilized and suspended with PBS at a concentration of 1 mg/mL. *G. vaginalis* (1.1 × 10^6^ colony forming unit (CFU)/mL) was inoculated in a 24-well plate with the BHIS broth, in which each 200 μL protein fraction was added. *G. vaginalis* was inoculated in the BHIS broth, in which 200 μL precipitated protein fraction (1 mg/mL) of MRS broth was added as control. The plate was incubated for 24 h at 37 °C under anaerobic condition (85% N_2_, 10% H_2_, and 5% CO_2_). After incubation, the suspension was spread on tryptic soy broth (TSB; BD) supplemented with 1.5% (w/v) bacto agar (BD), 5% (v/v) horse blood (Synergy Innovation Co., Ltd., Seongnam, Korea), 0.02% (v/v) menadione solution (BDH Chemicals Ltd., Poole, UK), and 1% (v/v) hemin solution (Sigma-Aldrich Co.). The agar plate was cultured for 48 h at 37 °C with 5% CO_2_ and colonies of *G. vaginalis* were counted.

### Evaluation of *G. vaginalis* biofilm formation

Inhibition of *G. vaginalis* biofilm formation with the < 3 kDa LCFSP was examined. The > 3 kDa LCFSP and < 3 kDa LCFSP were lyophilized and suspended in PBS at a concentration of 1 mg/mL. *G. vaginalis* (1.1 × 10^6^ CFU/mL) was inoculated in a 24-well plate with the BHIS broth, in which each 200 μL LCFSP fraction was added. *G. vaginalis* was inoculated in the BHIS broth, in which 200 μL precipitated protein fraction (1 mg/mL) of MRS broth was added as control. The plate was incubated at 37 °C for 24 h with 5% CO_2_. Biofilm was washed with 1 mL PBS, and then 1 mL 0.1% (v/v) crystal violet solution (Sigma-Aldrich Co.) was added to stain the biofilm. Staining was done by mixing the plate at 300 rpm for 15 min using well plate mixer (MX-M, DLAB, Riverside, CA, USA). Excess stain was removed, and stained biofilm was washed with 1 mL PBS and air-dried for 15 min. Biofilm was solubilized in 600 μL 33% (v/v) acetic acid, followed by mixing at 300 rpm for 10 min using the well plate mixer. OD was measured at 570 nm.

## Evaluation of ameliorative effect of *L. paracasei* CH88 and the < 3 kDa LCFSP on BV-induced mice

### Induction of BV and intravaginal treatment of *L. paracasei* CH88 and < 3 kDa LCFSP

Female C57BL/6 mice (7 weeks, 17–19 g) were purchased from Daehan Bio Link Co., Ltd. (Eumsung, Korea). The animals were housed in wire cages under a cycle of 12 h light/12 h dark at 50 ± 10% humidity and 23 ± 3 °C. All mice were fed AIN-93G diet (Research Diets Inc., New Brunswick, NJ, USA) and allowed to access water ad libitum. All animal experimental protocol was approved by Institutional Biosafety Committee of Seoul National University (SNUIBC-R201228-1; date of approval: December 31, 2020). All animal experiments were performed in accordance with a Guidelines for the Care and Use of Laboratory Animals issued by the Institutional Animal Care and Use Committee of Seoul National University (SNU-201228–2-1; date of approval: Feburary 1, 2021) and the ARRIVE guidelines.

BV was induced in accordance to the method of Jang et al.^29^ with minor modifications. After 1-week acclimatization, mice were divided into five groups with 6 mice each and all treatments were executed for 10 days. β-Estradiol 3-benzoate (0.5 mg; Sigma-Aldrich Co.) was diluted in 100 μL filter-sterilized sesame oil (Sigma-Aldrich Co.). The β-estradiol 3-benzoate solution was injected intraperitoneally 72 h before inducing BV and administered weekly thereafter^30^. On the day of BV induced, *G. vaginalis* (1.1 × 10^8^ CFU/20 μL PBS) was inoculated intravaginally. The control group was intravaginally treated with 20 μL PBS instead of *G. vaginalis* suspension. Each day for 6 days after infection, the control and *G. vaginalis*-infected group were intravaginally treated with 20 μL PBS. 100 μg metronidazole (Sigma-Aldrich Co.) was diluted in 20 μL sterilized PBS and injected intravaginally each day for 6 days after infection. *L. paracasei* CH88 (1.1 × 10^10^ CFU/20 μL PBS) was intravaginally injected each day for 6 days after infection. The lyophilized < 3 kDa LCFSP was diluted in sterilized PBS at a concentration of 1 mg/mL, 20 μL of which was then intravaginally injected each day for 6 days after infection. All mice were anesthetized with 4% isoflurane before intravaginal injection. Mice were euthanized with CO_2_ after 24 h of final administration. Vagina was washed with 100 μL sterilized PBS to obtain vaginal fluid and then excised. The excised vagina was fixed in 10% neutral buffered formalin solution (Sigma-Aldrich Co.) for histological examination and the other was stored at − 80 °C for enzyme-linked immunosorbent assay (ELISA) analysis.

### Evaluation of *G. vaginalis* CFU in vaginal fluid

Obtained vaginal fluid was directly transferred to anaerobic chamber with GasPak^TM^ EZ Anaerobe Container System (BD), and then serially 10-fold diluted. 100 μL of each diluted fluid was spread on TSB supplemented with 1.5% (w/v) bacto agar, 5% (v/v) horse blood, 0.02% (v/v) menadione solution, 1% (v/v) hemin solution, 4 mg/L gentamicin sulfate (Sigma-Aldrich Co.), 30 mg/L nalidixic acid (Sigma-Aldrich Co.), and 2 mg/L amphotericin B (Sigma-Aldrich Co.), followed by incubation for 48 h at 37 °C with 5% CO_2_. After the culture, colonies were counted. Colonies on TSB agar plate supplemented with antibiotics were confirmed to be *G. vaginalis* by gram-staining .

### Histopathological examination

Fixed vaginal tissue was stained with hematoxylin–eosin (H&E) and then assessed under a light microscope (× 200). H&E staining was performed by the Department of Clinical Laboratory Science, Wonkwang Health Science University (Iksan, Korea).

### Analysis of vaginal cytokine levels

ELISA kits (Thermo Fisher Scientific) were used to determine the levels of interleukin (IL)-1β, IL-6, IL-10, and tumor necrosis factor-α (TNF-α) in the vaginal tissue. 100 mg excised vagina was homogenized in 1 mL ice-cold RIPA lysis buffer containing 1% (v/v) protease inhibitor cocktail (Thermo Fisher Scientific) and 1% (v/v) phosphatase inhibitor cocktail (Thermo Fisher Scientific)^31^. Homogenates were centrifuged at 13,000 × g at 4 °C for 30 min and cytokine levels of the supernatants were examined by measuring OD at 450 nm.

### Statistical analysis

All statistical analyses were conducted using IBM SPSS Statistics 26.0 (Chicago, IL, USA). Results were represented as means and standard errors of the means (SEM). One-way analysis of variance (ANOVA) with Dunnett’s multiple comparison test (*p* < 0.05) or Duncan’s multiple range test (*p* < 0.05), and Kruskal–Wallis test with Dunn’s test (adjusted *p* < 0.05) were used to determine statistical significance between the groups.

## Results

**Figure 1:**
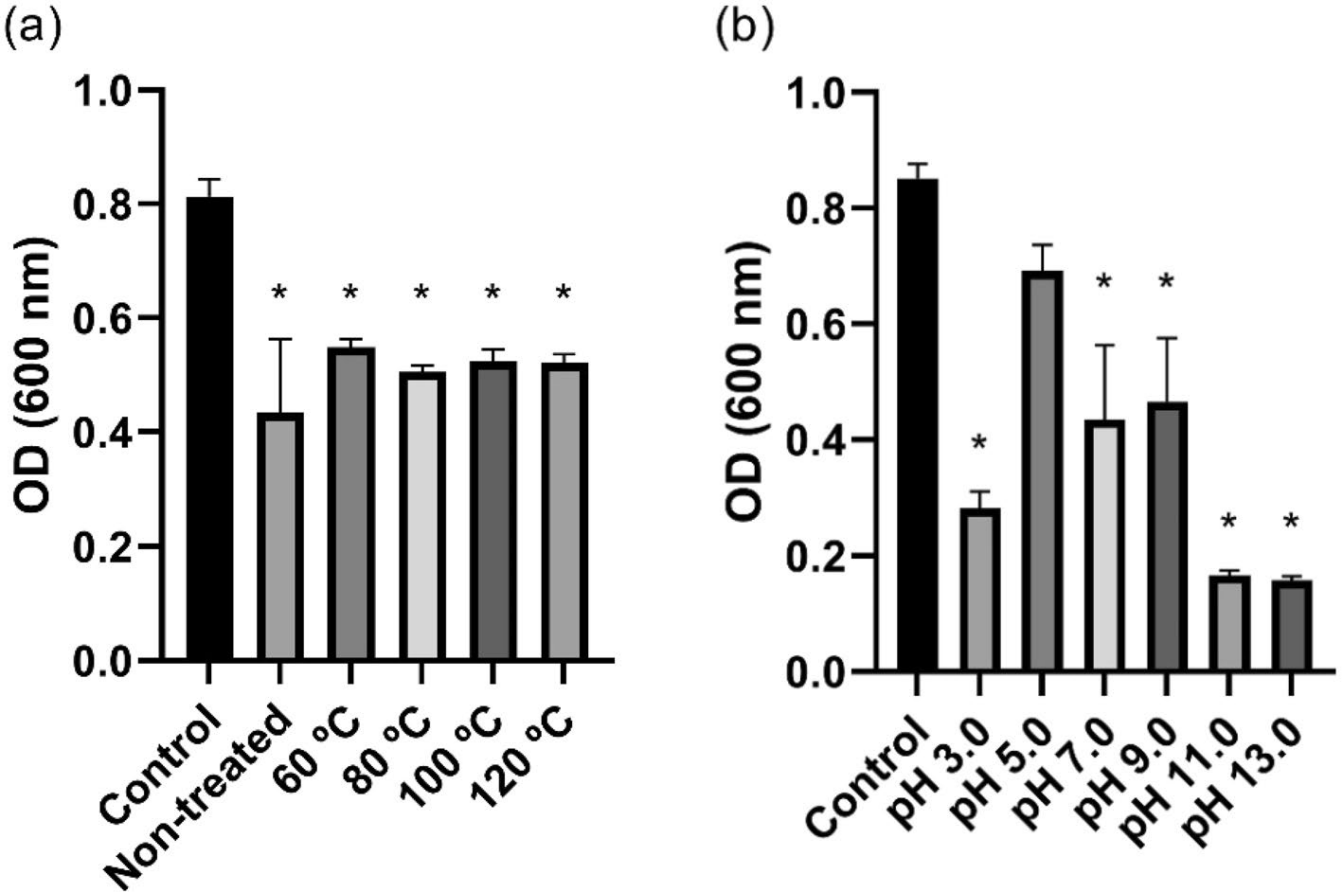
Optical density (OD) of *Gardnerella vaginalis* suspension incubated for 24 h with 5% (v/v) *Lactobacillus paracasei* CH88 cell-free supernatant treated at different temperatures for 30 min (**a**) and different pH (**b**). Bars are means and standard errors of the means (n = 3) of triplicate experiments. *Significant difference compared to the control (*p* < 0.05; one-way ANOVA with Dunnett’s multiple comparison test).

The viability of *G. vaginalis* was significantly reduced after 24 h incubation with the LCFS (Fig. 1a). Inhibitory activities of the LCFS against the growth of *G. vaginalis* depended on pH (Fig. 1b). The LCFS with pH 5.0 had the least activity. However, the growth curve of *G. vaginalis* treated with the LCFS with pH 5.0 exhibited delayed exponential phase compared to the control group, suggesting that the LCFS with pH 5.0 still had inhibitory effect (Supplementary Fig. 1b). The most active inhibition was observed in the LCFS with pH 11.0 and 13.0. These results suggest that antibacterial compound or compounds might be active potently at pH 11.0 and 13.0.

**Figure 2:**
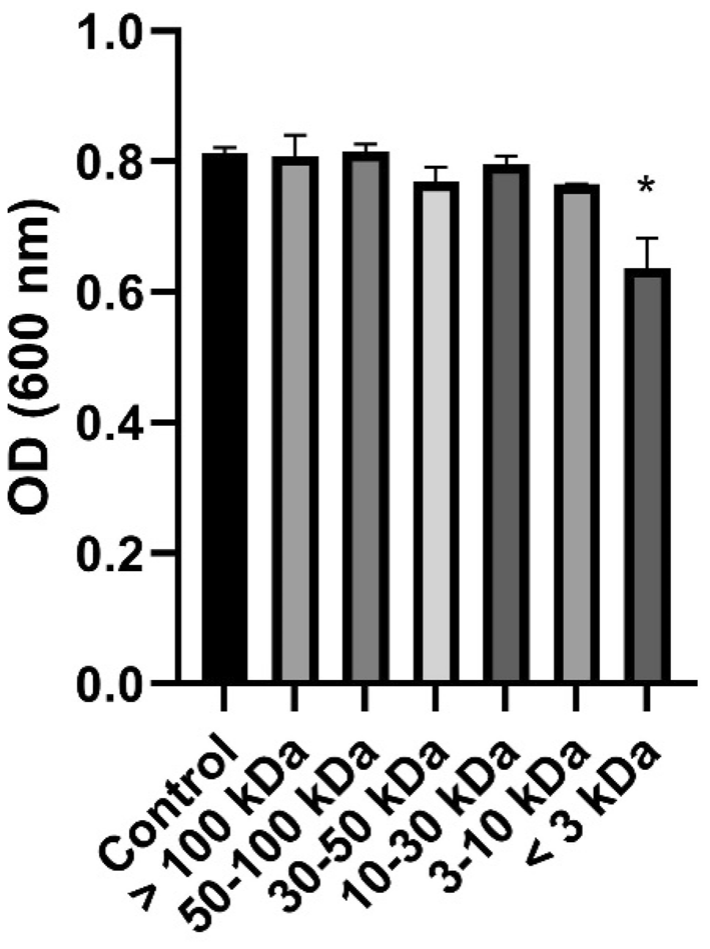
Optical density (OD) of *Gardnerella vaginalis* suspension incubated for 24 h with 5% (v/v) *Lactobacillus paracasei* CH88 cell-free supernatant fractions with different molecular weights. Bars are means and standard errors of the means (n = 3) of triplicate experiments. *Significant difference compared to the control (*p* < 0.05; one-way ANOVA with Dunnett’s multiple comparison test).

Among the fractions filtered with different MWCO membranes (< 3, 3–10, 10–30, 30–50, 50–100, and > 100 kDa), the < 3 kDa LCFS had the most antibacterial activity against *G. vaginalis* (Fig. 2). Furthermore, the < 3 kDa LCFS also delayed the exponential phase of *G. vaginalis* (Supplementary Fig. 2).

**Figure 3:**
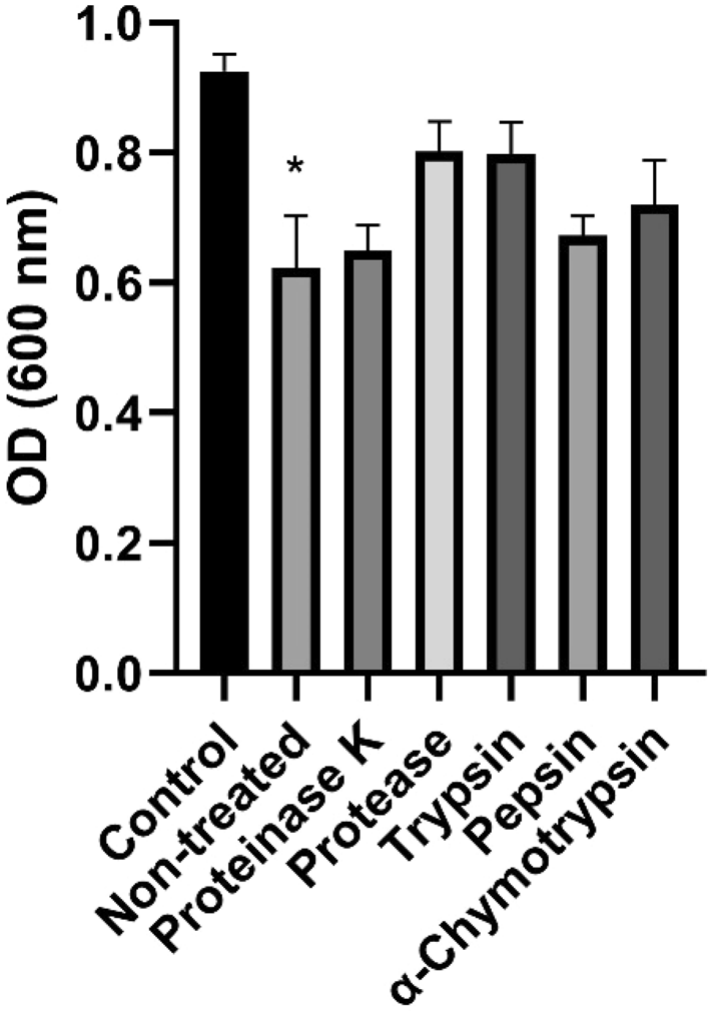
Optical density (OD) of *Gardnerella vaginalis* suspension incubated for 24 h with 5% (v/v) fraction below 3 kDa from *Lactobacillus paracasei* CH88 cell-free supernatant treated with different proteolytic enzymes. Bars are means and standard errors of the means (n = 3) of triplicate experiments. *Significant difference compared to the control (*p* < 0.05; one-way ANOVA with Dunnett’s multiple comparison test).

The < 3 kDa LCFS treated with protease, trypsin, and α-chymotrypsin showed a tendency to lose their antibacterial activity against *G. vaginalis* after 24 h incubation (Fig. 3). Among them, the < 3 kDa LCFS treated with trypsin or protease showed higher OD values in exponential phase than the others (Supplementary Fig. 3).

**Figure 4:**
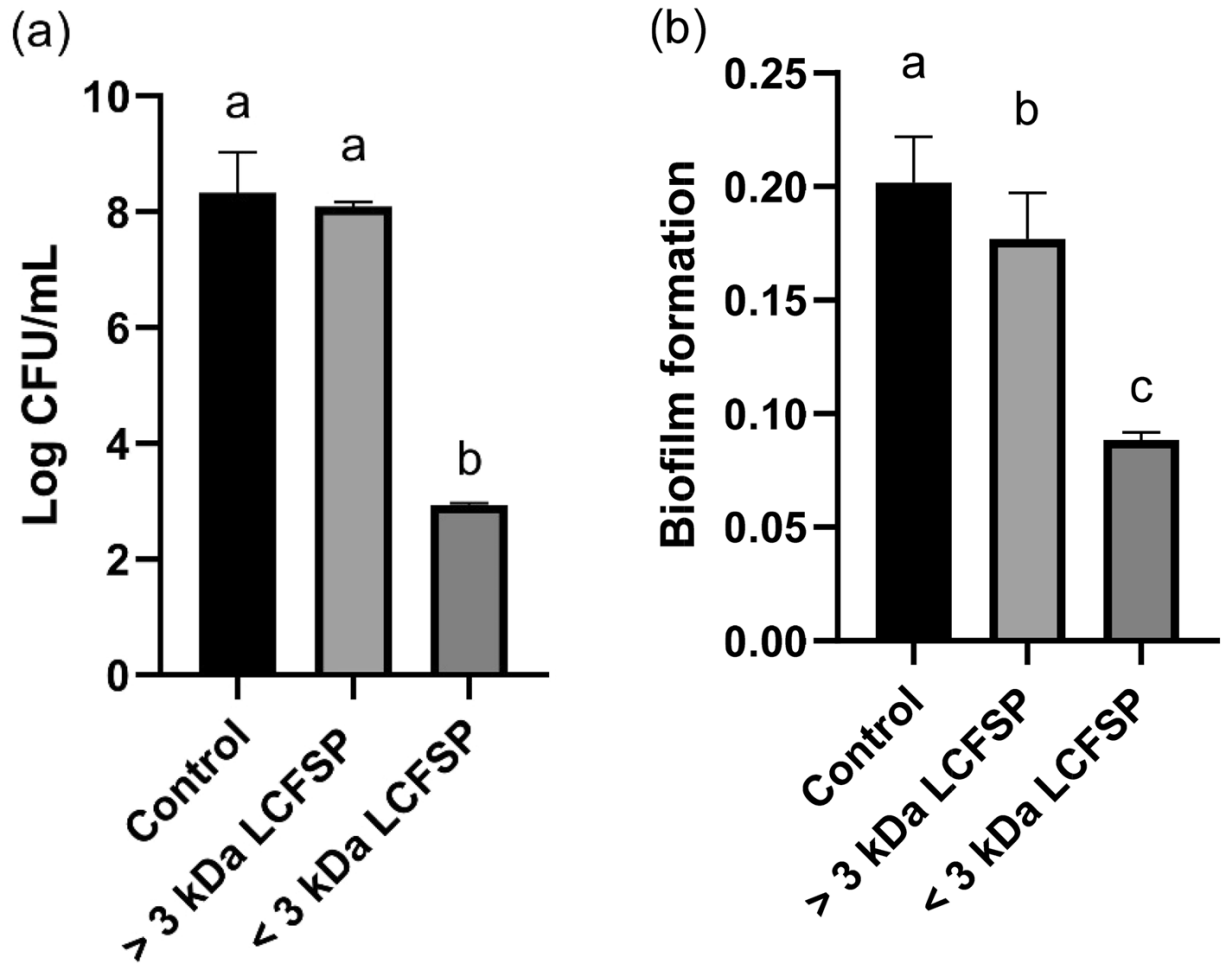
Colony forming unit (CFU) (**a**) and biofilm formation (**b**) of *Gardnerella vaginalis* treated with precipitated protein fraction of the *Lactobacillus paracasei* CH88 cell-free supernatant (LCFSP; 1 mg/mL). Bars are means and standard errors of the means (n = 3) of triplicate experiments. Different small letters indicate significant differences (*p* < 0.05; one-way ANOVA and Duncan’s multiple range test). (*p* < 0.05; one-way ANOVA and Duncan’s multiple range test).

We investigated whether protein precipitates from the LCFS, including the > 3 kDa LCFSP and the < 3 kDa LCFSP, had antibacterial activity against *G. vaginalis*. The CFU of *G. vaginalis* in the < 3 kDa LCFSP-treated group was significantly lower than the control and > 3 kDa LCFSP-treated groups (*p* < 0.05) (Fig. 4a). Both of the LCFSP reduced the biofilm formation of *G. vaginalis* compared to the control group (Fig. 4b). Especially, the < 3 kDa LCFSP significantly more inhibited the biofilm formation than the > 3 kDa LCFSP (*p* < 0.05).

**Figure 5:**
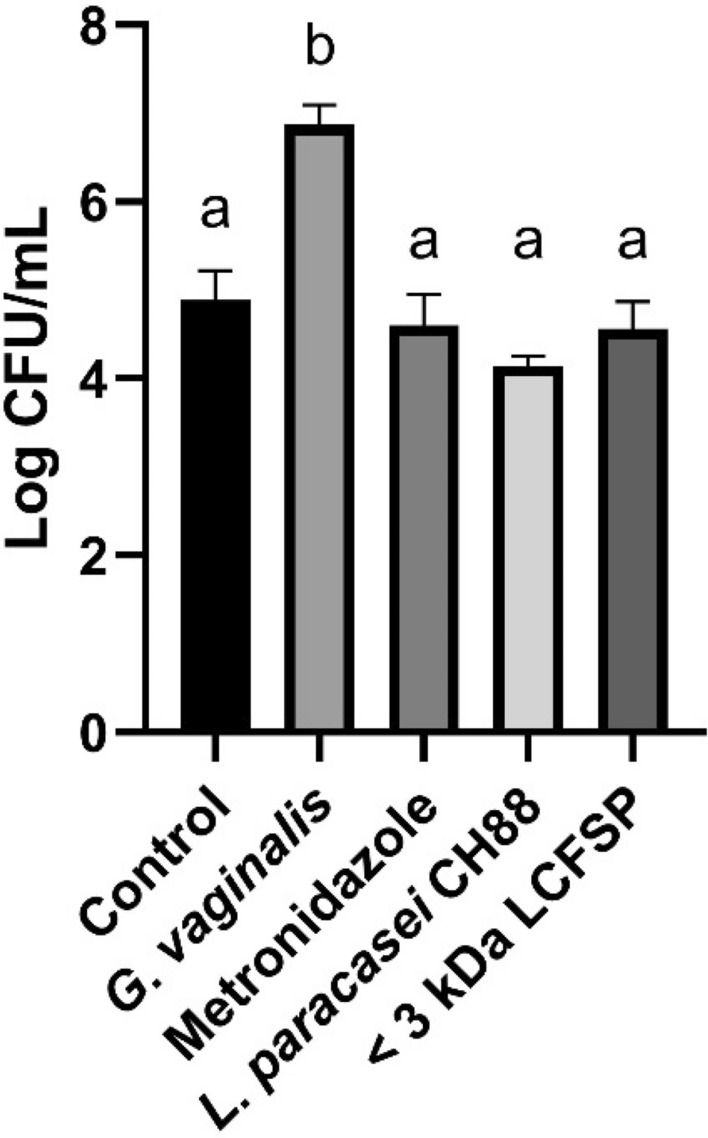
Colony forming unit (CFU) of *Gardnerella vaginalis* in vaginal fluid of the mice intravaginally treated with phosphate-buffered saline (20 μL), *G*. *vaginalis* (1 × 10^8^ CFU), metronidazole (100 μg), *Lactobacillus paracasei* CH88 (1 × 10^10^ CFU), and protein fraction below 3 kDa of *L. paracasei* CH88 cell-free supernatant (20 μg; < 3 kDa LCFSP). Bars are means and standard errors of the means (n = 6). Different small letters indicate significant differences (*p* < 0.05; one-way ANOVA and Duncan’s multiple range test).

**Figure 6:**
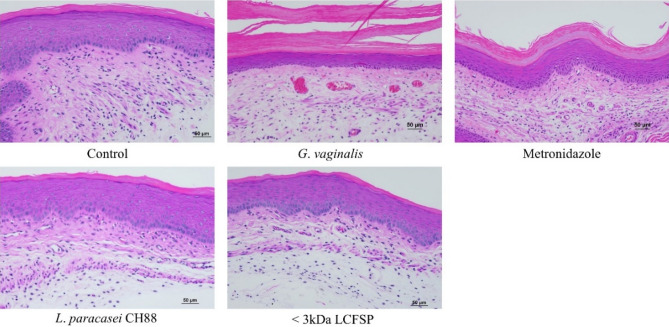
Hematoxylin–eosin staining of the murine vaginal tissue intravaginally treated with phosphate-buffered saline (20 μL), *G*. *vaginalis* (1 × 10^8^ CFU), metronidazole (100 μg), *Lactobacillus paracasei* CH88 (1 × 10^10^ CFU), and protein fraction below 3 kDa of *L. paracasei* CH88 cell-free supernatant (20 μg; < 3 kDa LCFSP).

**Figure 7:**
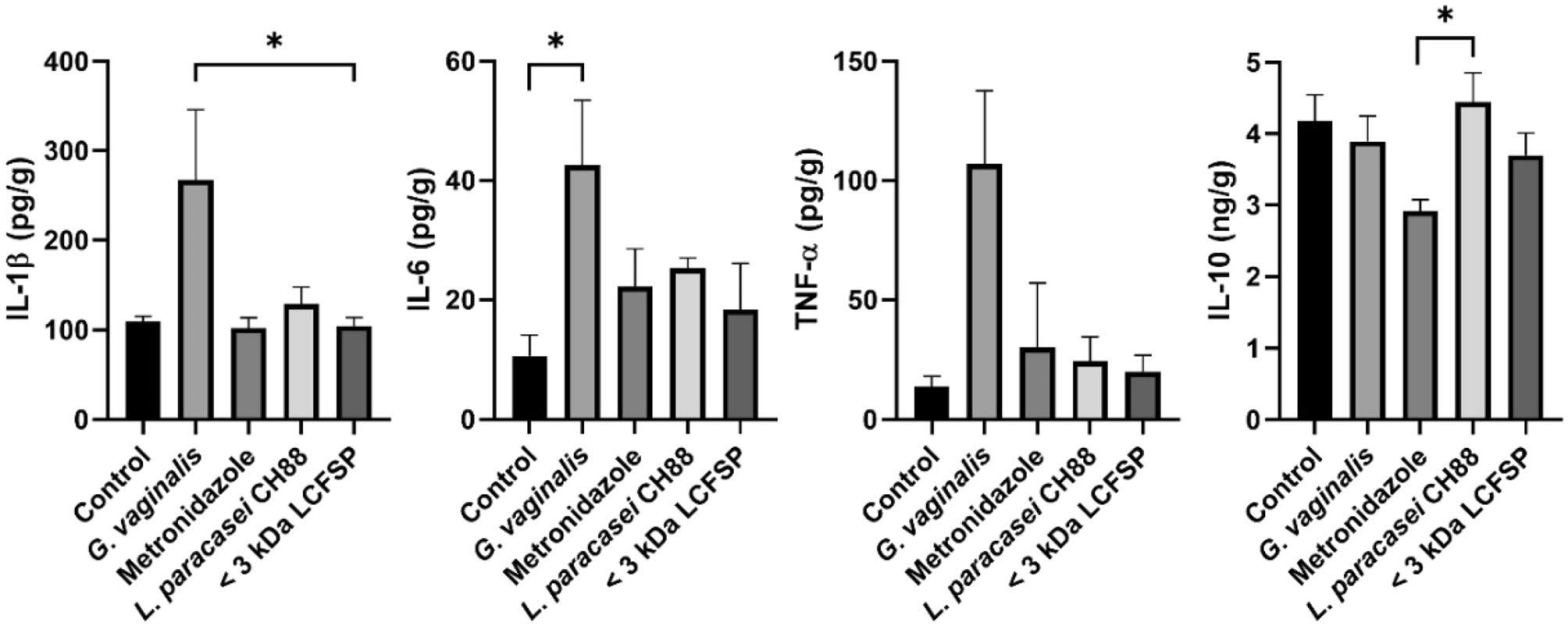
Cytokines in the murine vagina intravaginally treated with phosphate-buffered saline (20 μL), *G*. *vaginalis* (1 × 10^8^ CFU), metronidazole (100 μg), *Lactobacillus paracasei* CH88 (1 × 10^10^ CFU), and protein fraction below 3 kDa of *L. paracasei* CH88 cell-free supernatant (20 μg; < 3 kDa LCFSP). Bars are means and standard errors of the means (n = 6) of duplicate experiments. *Significant difference (adjusted *p* < 0.05; Kruskal–Wallis test with Dunn’s test).

The CFU of *G. vaginalis* were significantly reduced in vaginal fluid of the mice to which *L. paracasei* CH88, < 3 kDa LCFSP, or metronidazole was intravaginally administered (Fig. 5). Vaginal tissue of the *G. vaginalis* group showed increased exfoliation of vaginal epithelial cells and thickness of transitional epithelium in H&E staining (Fig. 6). On the other hand, the *L. paracasei* CH88 or < 3 kDa LCFSP group showed less vaginal epithelial cell exfoliation and thinner transitional epithelium than the *G. vaginalis* group. Intravaginal treatment of *L. paracasei* CH88 or the < 3 kDa LCFSP tended to reduce the levels of IL-1β, IL-6, and TNF-α in vaginal tissue of the mice (Fig. 7). IL-10 production was higher in the CH88 group than in the *G. vaginalis* and metronidazole groups.

## Discussion

Antibacterial effect of cell-free supernatant fractions of *L. paracasei* CH88 against *G. vaginalis* was investigated and characterized in-vitro and in-vivo. The LCFS still had antibacterial activity after thermal treatment at the various temperatures, indicating that antibacterial compound or compounds in the LCFS were stably active even when treated at 120 °C for 30 min (Supplementary Fig. 1a). Bacteriocin fraction from *L. paracasei* SD1 cell-free supernatant lost their activity completely after thermal treatment at 120 °C for 20 min^32^. However, *L. paracasei* HD1-7 cell-free supernatant maintained their activity after thermal treatment at 121 °C for 20 min^33^. Class II bacteriocin, one of food preservatives, is known to be stable during thermal food processing^34^. The LCFS showed the highest antibacterial activity against *G. vaginalis* at pH 11.0 and pH 13.0 in this study. Antibacterial activity of *L. paracasei* ZFM54 bacteriocin was significantly reduced at pH 5.0 [e], which was consistent with our result. However, bacteriocin fraction from *L. paracasei* SD1 cell-free supernatant showed the highest antibacterial activity at pH 5.0 and 6.0^32^. The most active inhibition of *L. paracasei* HD1-7 cell-free supernatant was observed at pH 3.0, but its antibacterial activity was inactivated at pH 9.0^33^.

The < 3 kDa LCFS had the most antibacterial activity against *G. vaginalis* in this study, suggesting that the antibacterial compound or compounds in the LCFS pass through 3 kDa MWCO membrane. Antibacterial compounds below 10 kDa, such as class II bacteriocins and bacteriocin from *Lactobacillus* spp., have been reported to easily permeate cell wall causing cell wall leakage^35–37^, leading to irreversible alteration of the cellular membrane.

After proteolytic enzyme treatment on the < 3 kDa LCFS, antibacterial activity against *G. vaginalis* was reduced. These results suggest that antibacterial compound or compounds in the < 3 kDa LCFS might be mainly composed of proteins and easily hydrolyzed by trypsin or protease, which prevents its accumulation in body unlike antibiotics^38^.

Among the LCFSP, < 3 kDa LCFSP had antibacterial activity in this study. Numerous studies identified inhibitory activity of protein precipitates secreted by various bacteria. Protein precipitates from cell-free supernatant of *L. acidophilus* KS400, *Bacillus amyloliquefaciens*, and *B. coagulans* had inhibitory activities against the growth of *G. vaginalis* and their molecular weights ranged from 3 kDa to 7.5 kDa^39–41^. In this study, antibiofilm compound or compounds in the LCFSP mainly existed in the < 3 kDa LCFSP. *G. vaginalis* develops a dense biofilm adherent to vaginal epithelium in the women who suffer from BV^42^. Biofilm offers *G. vaginalis* a favorable environment providing nutrients and protecting it from antimicrobial agents^43^. Therefore, preventing the biofilm formation of *G. vaginalis* might be a primary strategy to treat BV and to prevent its recurrence^43^. However, it was reported that antibiotics such as metronidazole and tobramycin have no significant disruptive effect on *G. vaginalis* biofilm in-vitro^44^. Saunders et al. reported that *L. reuteri* RC-14 and *L. rhamnosus* GR-1 could reduce *G. vaginalis* biofilm, suggesting that biosurfactants produced by these lactobacilli might contribute to inhibiting biofilm formation^45^. It was also reported that *L. delbrueckii* DM8909, *Lactiplantibacillus* plantarum ATCC14917, and *Lactiplantibacillus plantarum* ZX27 had antibiofilm abilities against *G. vaginalis*^46^. It can be concluded that the < 3 kDa LCFSP might have a potency to prevent the formation of *G. vaginalis* biofilm. However, further study should be performed to identify antibiofilm agent(s) in the < 3 kDa LCFSP.

Intravaginal treatment of metronidazole, *L. paracasei* CH88, and < 3 kDa LCFSP significantly reduced CFU of *G. vaginalis* in vaginal fluid. Previous studies also reported that intravaginal and oral administration of lactobacilli such as *L. johnsonii* HY7042, *L. rhamnosus* HN001, and *L. acidophilus* GLa-14 could ameliorate BV via reducing *G. vaginalis* level in vagina^29,31^. Jang et al. reported that intravaginal administration of cell-free supernatant from *L. fermentum* SNUV175 and *L. crispatus* SNUV220 had ameliorative effects on vulvovaginal candidiasis in murine model by reducing the CFU of *Candida albicans*, which is also known as a representative pathogenic yeast causing vaginal disorder^47^. Lactobacilli have been known to be able to inhibit the growth of *G. vaginalis* by producing antimicrobial compounds as well as lactic acid^23,24^. Since pH of the < 3 kDa LCFSP was set at 7.0, it could be presumed that antibacterial activity of *L. paracasei* CH88 against *G. vaginalis* might be mainly attributed to unidentified compounds in the < 3 kDa LCFSP. Intravaginal treatment of *G. vaginalis* increased thickness of vaginal transitional epithelium in this study. Increased thickness of transitional epithelium in vagina was reported to be related to epithelial proliferation, which results in cell exfoliation^48^. Exfoliated vaginal epithelial cells covered with adherent anaerobic bacteria have been commonly observed in vaginal fluid of the women who have BV, but not in the women who do not^49^. Our results suggest that *L. paracasei* CH88 or the < 3 kDa LCFSP reduced *G. vaginalis*-induced vaginal epithelial cell exfoliation. Intravaginal inoculation of *G. vaginalis* was reported to induce immune response, resulting in elevating the levels of IL-1β, IL-6, IL-8, and IL-10 in cervicovaginal fluid^50^. *L. paracasei* CH88 and < 3 kDa LCFSP-treated group showed lower IL-1β, IL-6, TNF-α level compared to *G. vaginalis* group. Previous studies also reported that intravaginal and oral administration of *L. johnsonii* HY7042, *L. rhamnosus* HN001, and *L. acidophilus* GLa-14 tended to reduce the levels of IL-1β, IL-6, and TNF-α, while increasing IL-10 production in mice with *G. vaginalis*-induced BV^29,31^, which were consistent with our results.

## Conclusion

The antibacterial effect of *L. paracasei* CH88 may be due to unidentified proteins in the fraction below 3 kDa of *L. paracasei* CH88 cell-free supernatant. *L. paracasei* CH88 or < 3 kDa LCFSP could ameliorate BV via not only inhibiting the growth of *G. vaginalis* and its biofilm formation, but also reducing exfoliation of vaginal cells and production of pro-inflammatory cytokines. However, this study has some limitations that in-vitro and in-vivo antibacterial experiments against *F. vaginae* and *P. bivia* should be followed since BV is a polymicrobial infection. Moreover, safety assessment must be followed to apply this treatment to humans.

## Supplementary Information

Supplementary Information 1.

Supplementary Information 2.

Supplementary Information 3.

## Data Availability

The data presented in this study are available on reasonable request and for non-commercial purposes.
