# Supplementary material for: Antibacterial effect of cell-free supernatant fraction from Lactobacillus paracasei CH88 against Gardnerella vaginalis
Source: Sci Rep. 2022 Mar 19;12:4763. doi: 10.1038/s41598-022-08808-7 (PMC8934363; doi:10.1038/s41598-022-08808-7)
Supplement: Supplementary file 1 — Supplementary Information 1. [file 41598_2022_8808_MOESM1_ESM.docx]

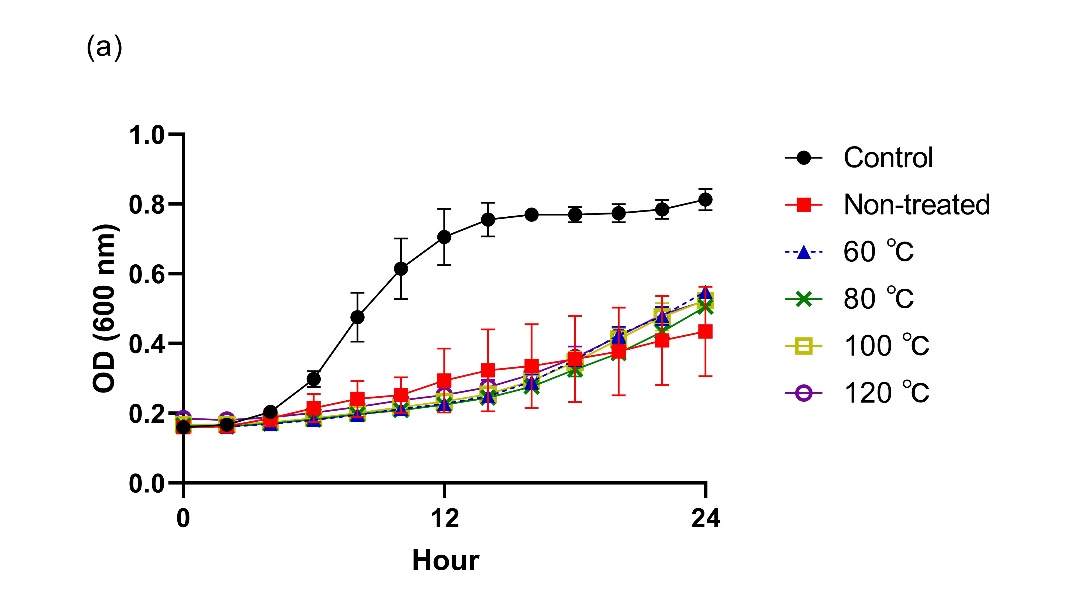

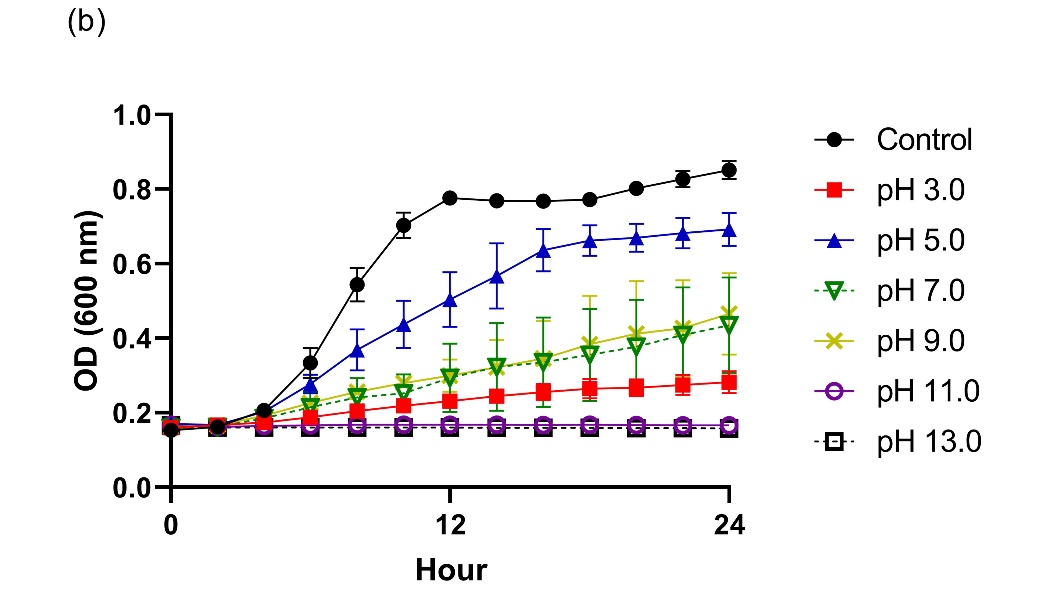


Supplementary Fig. 1 Growth curves of *Gardnerella vaginalis* suspensions incubated for 24 h with 5% (v/v) *Lactobacillus* *paracasei* CH88 cell-free supernatant treated at different temperatures for 30 min (a) and different pH (b). Data points are means ± standard errors of the means (n=3; error bars). Some error bars lie within the data points. OD, optical density.
