# Supplementary material for: Antibacterial effect of cell-free supernatant fraction from Lactobacillus paracasei CH88 against Gardnerella vaginalis
Source: Sci Rep. 2022 Mar 19;12:4763. doi: 10.1038/s41598-022-08808-7 (PMC8934363; doi:10.1038/s41598-022-08808-7)
Supplement: Supplementary file 3 — Supplementary Information 3. [file 41598_2022_8808_MOESM3_ESM.docx]

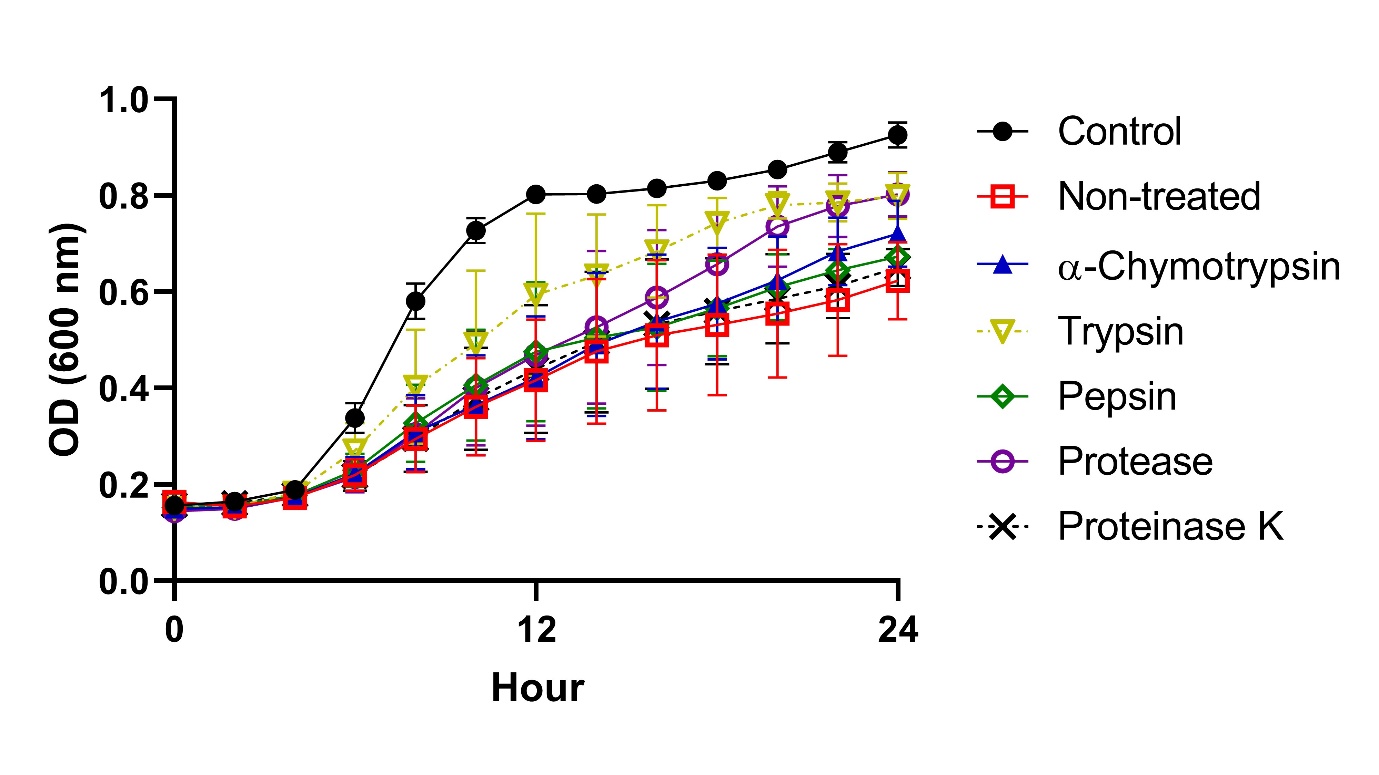
 Supplementary Fig 3. Growth curves of *Gardnerella vaginalis* suspensions incubated for 24 h with 5% (v/v) fraction below 3 kDa from *Lactobacillus* *paracasei* CH88 cell-free supernatant treated with different proteolytic enzymes. Data points are means ± standard errors of the means (n=3; error bars). Some error bars lie within the data points. OD, optical density.
